# Standardization of *Cassia spectabilis* with Respect to Authenticity, Assay and Chemical Constituent Analysis

**DOI:** 10.3390/molecules15053411

**Published:** 2010-05-10

**Authors:** Angeline Torey, Sreenivasan Sasidharan, Chen Yeng, Lachimanan Yoga Latha

**Affiliations:** Institute for Research in Molecular Medicine (INFORMM), Universiti Sains Malaysia, 11800 USM, Penang, Malaysia

**Keywords:** *Cassia spectabilis*, standardization, microscopy, medicinal plants

## Abstract

Quality control standardizations of the various medicinal plants used in traditional medicine is becoming more important today in view of the commercialization of formulations based on these plants. An attempt at standardization of *Cassia spectabilis* leaf has been carried out with respect to authenticity, assay and chemical constituent analysis. The authentication involved many parameters, including gross morphology, microscopy of the leaves and functional group analysis by Fourier Transform Infrared (FTIR) spectroscopy. The assay part of standardization involved determination of the minimum inhibitory concentration (MIC) of the extract which could help assess the chemical effects and establish curative values. The MIC of the *C. spectabilis* leaf extracts was investigated using the Broth Dilution Method. The extracts showed a MIC value of 6.25 mg/mL, independent of the extraction time. The chemical constituent aspect of standardization involves quantification of the main chemical components in *C. spectabilis*. The GCMS method used for quantification of 2,4-(1*H*,3*H*)-pyrimidinedione in the extract was rapid, accurate, precise, linear (R^2^ = 0.8685), rugged and robust. Hence this method was suitable for quantification of this component in *C. spectabilis.* The standardization of *C. spectabilis* is needed to facilitate marketing of medicinal plants, with a view to promoting the export of valuable Malaysian Traditional Medicinal plants such as *C. spectabilis*.

## 1. Introduction 

Herbal medicines are the therapeutic experiences of generations of practicing physicians of traditional medicine over hundreds of years and they are known to be oldest health care products that have been used by mankind all over the world to treat various types of ailments [1]. Herbal traditional medicine has gained considerable momentum worldwide during the past decade. An important factor, which can contribute to the consistent quality of herbal products, is to have adequate standardization. Due to the natural heterogeneity, the quality of herbal starting materials obtained from wild collections shows great fluctuations. Thus, standardization of herbal products has been extensively promoted during the last years. Standardization is defined by the American Herbal Products Association as “… the body of information and controls necessary to produced materials of reasonable consistency. This is achieved through minimizing the inherent variation of natural product composition through quality assurance practices applied to agricultural and manufacturing processes”[2]. For standardization and quality assurance purposes, the following three attributes are must be verified: authenticity, purity and assay [3]. Hence, in this paper we report an attempt for the standardization of *Cassia spectabilis* leaf with respect to authenticity, assay and chemical constituent analysis.

The genus *Cassia*, comprising about 600 species widely distributed worldwide is well known for its diverse biological and pharmacological properties [4]. *Cassia spectabilis* (sin *Senna spectabilis*) (DC) Irwin et Barn (Leguminosae) is widely grown as an ornamental plant in tropical and subtropical areas, and has been commonly used in traditional medicine for many years. It has also been used in traditional Brazilian medicine for the treatment of flu and cold, as a laxative and purgative [5]. Other actions like antifungal [6], antibacterial [7], and antioxidant [8] have been reported. The plant is collected from the wild sources and varies in constituents and efficacy due to its geographical diversity. Improper collection and storage condition lead to the deterioration of the raw material. Keeping in view the abovementioned problems, it was important to standardize the leaves of *C. spectabilis* to establish a quality and identity profile of the plant for the purpose of overall quality assurance of this medically important plant. Since there are no reports in the literature regarding the standardization of *C. spectabilis* leaves, in the present investigation an attempt has been made to standardize them by using macroscopic and microscopic characteristics, authenticity, biological activity assay and chemical constituent analysis. 

## 2. Results and Discussion

### 2.1. Herbarium

**Figure 1:**
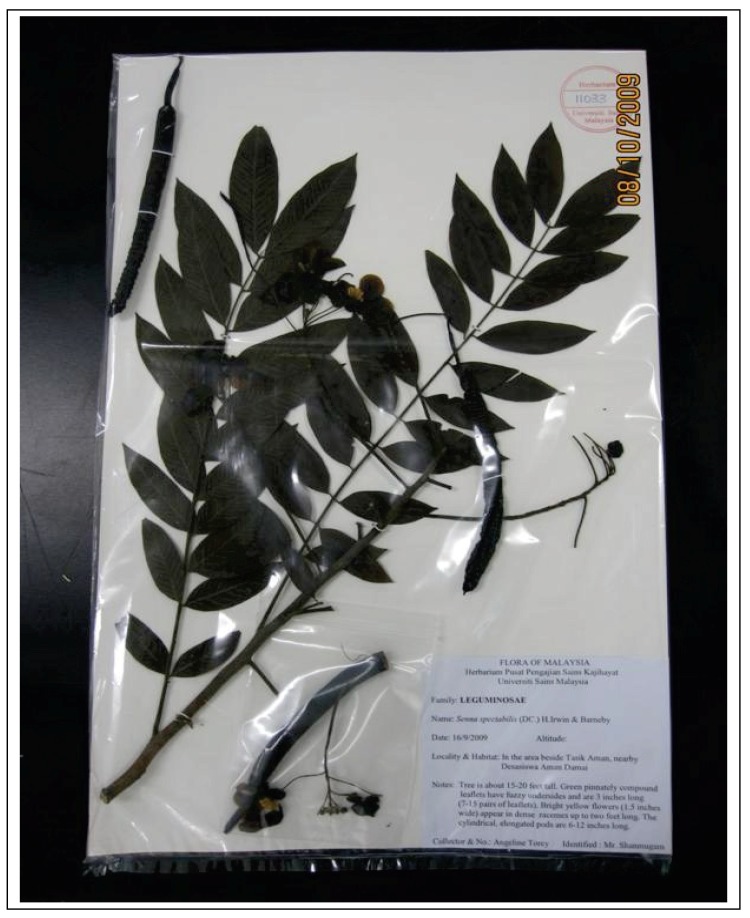
The *Cassia spectabilis* herbarium.

Since, plant classifications are constantly changing, the verification of the authenticity of the specimens using the herbarium is becoming an increasingly important step. Voucher specimens help cross-reference these changes to previous plant materials. Hence, in this study a *C. spectabilis* herbarium was prepared (Figure 1). *C. spectabilis* is a recognized source of useful chemicals for the pharmaceutical industries, but authenticity of specimens is important for these industries to avoid the acceptance of wrong plant materials for extracting actives, potentially resulting in a loss of billions of dollars. Therefore, the correctly identified herbarium specimens can be used for comparison of future *C. spectabilis* collections. In addition, using a herbarium specimen will allow the study of morphological or anatomical details that will help identify field collected plant specimens in the sterile or non-reproductive condition. 

### 2.2. Macroscopy and microscopy of leaf

**Figure 2:**
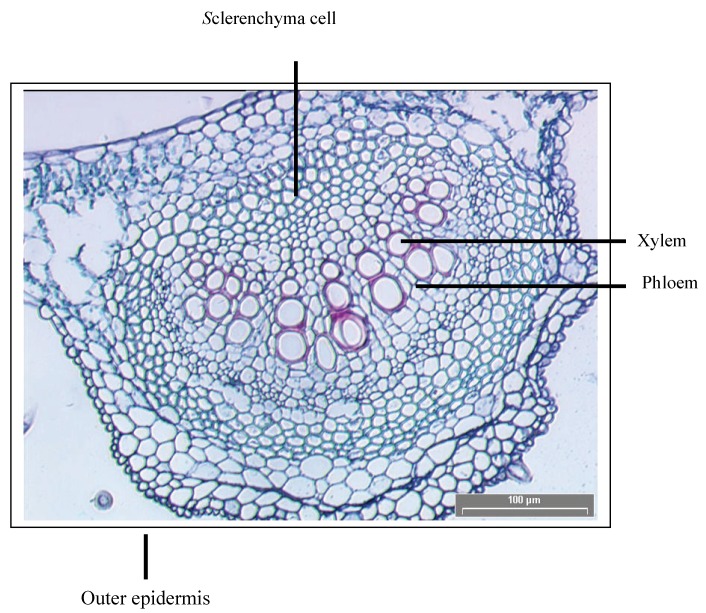
Transverse section of the leaf of *C. spectabilis.*

Most of the regulatory guidelines and pharmacopoeias suggest macroscopic and microscopic evaluation of the botanical materials for standardization [9]. Thereby, in this study a macroscopic and microscopic evaluation of the leaf material was done as the leaf extract was to be used in our subsequent anticandidal biofilm activity study. The leaves of *C. spectabilis* were subjected to macroscopical examination and observations were recorded. The proper examination of the leaves was carried out under sunlight and artificial light sources similar to daylight. The leaves of *C. spectabilis* were observed to be green, pinnate leaves, 7–15 pairs of leaflets, 2 to 3.6 inches long (5–9 cm), entire margin with fuzzy undersides. A photomicrograph of the identifying features of the leaf is shown in Figure 2. Transverse section of the leaf of *C. spectabilis* through the midrib revealed the presence of upper epidemis with straight anticlinal walls and elongated palisade cells underneath. There are conspicuous spongy parenchymal cells on the lower surface and in between were the xylem and phloem vessels.

### 2.3. Fourier Transform Infrared (FTIR) fingerprinting

**Figure 3:**
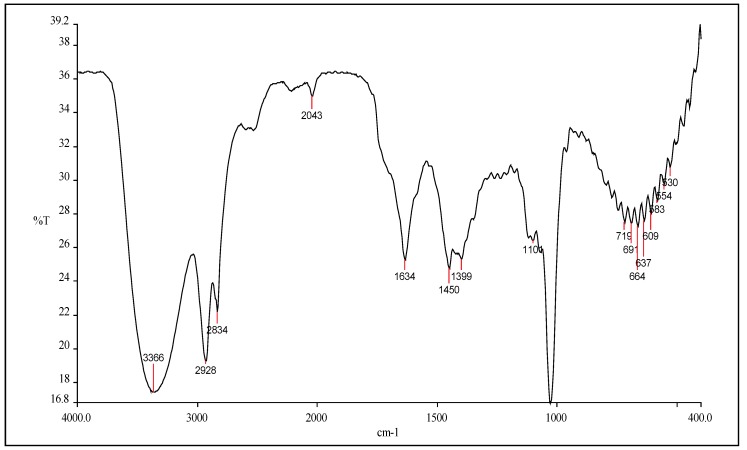
FTIR spectra of methanolic extract of *Cassia spectabilis* (KBr disc), cm^-1^.

The use of FTIR fingerprinting for herbal extract tends to focus on identification and assessment of the stability of the chemical constituents’ functional groups as observed by FTIR analysis. The results of FTIR fingerprint for the methanolic extract is shown in Figure 3. The results of functional group analysis using FTIR demonstrated that the existence of various characteristic functional groups in the *C. spectabilis* extract (Figure 3). Eight major peaks in the range 700–1,200 cm^-1^, 1,139–2,000 cm^-1^ and 2,043–4,000 cm^-1^ were observed in the FTIR spectra. Therefore, for future *C. spectabilis* methanolic extraction this FTIR spectrum can be used for comparison. Obviously, the FTIR fingerprint can be used to ensure that the functional groups in the new extract are present in reproducible manner although a new extraction has been done. As a result, FTIR fingerprints assist the manufacturer in controlling and assuring the consistency and the standard quality of the extract in each phase of an extraction.

### 2.4. Minimum inhibitions concentration (MIC)

Consistency in the biological activity is an essential requirement for the effective use of therapeutic agents.This consistency ensures that the extract retains its therapeutic parameters throughout the shelf life assigned to the extract. Hence, studies that confirm that the extract retains its efficacy and biological activity are crucially important before it’s use by the pharmaceutical industries. Hence, in this study the antifungal activity was used for this purpose by determining the MIC value. The MIC of the *C. spectabilis* leaves extracts was investigated by using Broth Dilution Method against pathogenic yeast *Candida albicans*. The extracts showed a MIC value of 6.25 mg/mL and retained the value for different extraction times (Table 1).

### 2.5. Chemical constituent standardization

**Figure 4:**
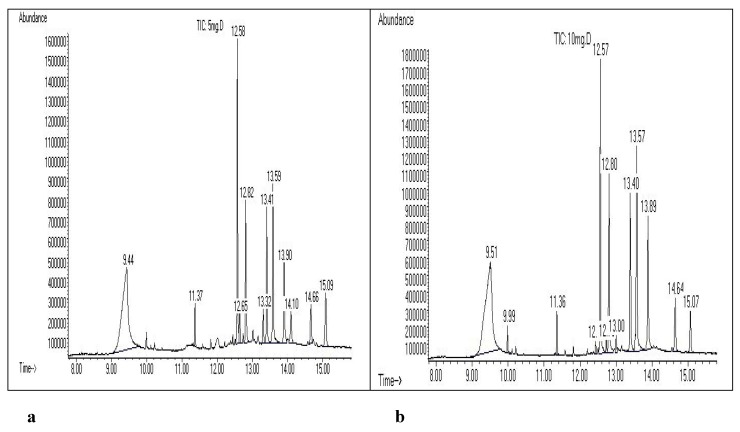

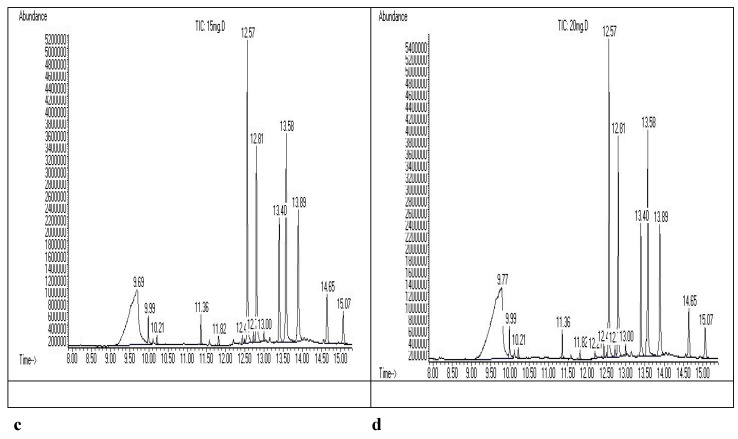
GCMS profile of 2,4-(1*H*,3*H*)-pyrimidinedione at retention time 12.57. a = 5 mg/mL, b = 10 mg/mL, c = 15 mg/mL, d = 20 mg/mL.

**Figure 5:**
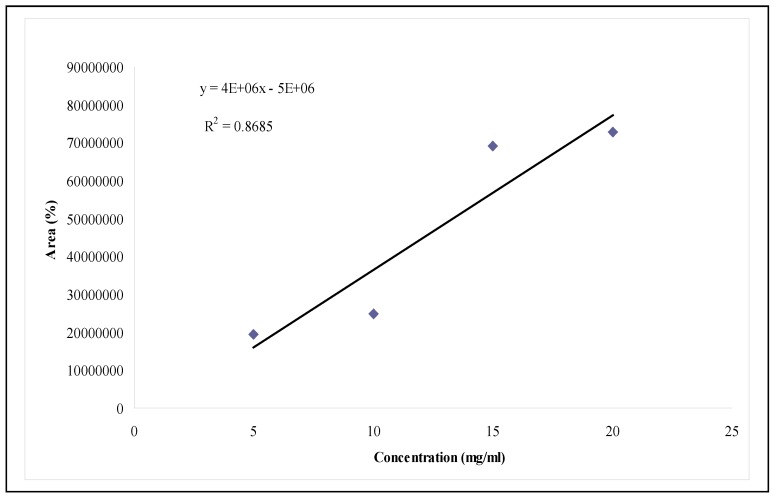
Calibration curve of 2,4-(1*H*,3*H*)-pyrimidinedione by GCMS.

Along with authentication of species identity and assay, chemical constituent standardization is also required for quality control in the use of plant materials for pharmaceutical purposes. In this study the *C. spectabilis* leaf extract was identified as an extract containing no constituents documented as being determinant or relevant for efficacy, or as having any pharmacological or clinical relevance. In this case, chemically defined constituent (markers) without known therapeutic activity may be used for control purposes [1]. These markers may be used to monitor good manufacturing practice or as an indication for the content of the extract [1]. A standard curve was plotted using the area of the peak obtained in the GCMS chromatogram against the concentration of 2,4-(1*H*,3*H*)-pyrimidinedione. The GCMS profile and calibration curve of the compound is given in Figure 4 and Figure 5. This could be applied for the standardization and validation of *C. spectabilis* leaf extract in terms of 2,4-(1*H*,3*H*)-pyrimidinedione as a chemical marker [10]. According to Lazarowych and Pekos [10], chemical and chromatographic techniques are currently used for identification and assessment of chemical constituents of medicinal plants. The most common techniques are high performance liquid chromatography (HPLC), thin layer chromatography (TLC), and gas chromatography (GC) [10]. Chromatographic fingerprints and marker compounds are used as reference standards, which indicate the purity, identity, and quality of the herbal drug [10].

## 3. Experimental

### 3.1. Chemicals and reagents 

All the chemicals and reagents used were of analytical grade, purchased from Sigma Chemical Co. (St Louis, MO, USA) or Merck (Darmstadt, Germany). 

### 3.2. Plant sample

A sample of *C. spectabilis* was collected in April 2009, from Universiti Sains Malaysia campus, Pulau Pinang, Malaysia and authenticated at the Herbarium of the School of Biological Sciences, Universiti Sains Malaysia, Pulau Pinang, Malaysia where a sample (voucher number 11033) has been deposited.

### 3.3. Qualitative investigation

The macroscopic features of the fresh leaves were determined using the methods of Evans [11]. Anatomical sections, a surface preparation of the fresh leaves sample for the microscopy was carried out according to methods outlined by Brain and Turner [12]. Morphological examination of the *C. spectabilis* leaf specimen was used to identify the leaf structures. Leaf tissue was fixed in 10% buffered formalin. After fixation, the tissue was dehydrated in a graded series of alcohols, cleared in xylene and embedded in paraffin wax. Multiple 5 mm sections from the block were mounted on slides and stained with 0.5% methylene blue and examined under a light microscope.

### 3.4. FTIR analytical methods

The methanol extracts of *C. spectabilis* was mixed with KBr salt, using a mortar and pestle, and compressed into a thin pellet. Infrared spectra were recorded on a Shimadzu FTIR Spectrometer 8000 series, between 4,000–500 cm^-1^. All determinations were performed in triplicate [13].

### 3.5. Determination of MIC of leave extract

The serial tube dilution technique was used to determine the MIC of the extract against *Candida albicans*. A stock solution of the plant extract was prepared to give a concentration of 200 mg/mL. In a serial dilution technique, 5 mL of the prepared stock solution was transferred to a test tube containing 5 mL of potato dextrose broth medium to give concentration of 100 mg/mL, from which 5 mL was transferred to another test tube containing 5 mL of potato dextrose broth medium to give concentrations ranging from 100 mg/mL to 0.1 mg/mL. After the preparation of the *Candida albicans* suspensions (10^7^ organisms per mL), 500 µL of the yeast suspensions was added to each broth dilution. After 24 h incubation at 37 °C, the tubes were examined for growth. The MIC of the extract was taken as the lowest concentration that shows no growth. Growth was observed in those tubes where the concentration of the extract is below the inhibitory level and the broth medium was turbid (cloudy) when observed [14]. 

### 3.6. Gas chromatography–mass spectrometry (GC–MS) analysis

The GC/MS analysis was done on a thermo gas chromatograph - mass spectrometer (model Shimadzu 2010) equipped with DB-5 capillary column (30 m long. 0.25 mm i.d., film thickness 0.25 μm). The column temperature program was 50 °C for 6 min, with 5 °C per min increases to 250 °C; which was maintained for 30 min. The carrier gas was helium at a flow rate of 1 mL/min (splitless mode). The detector and injector temperatures were both maintained at 250 °C. The quadrupole mass spectrometer was scanned over the range 28–400 amu at 1 scan s^-1^, with an ionising voltage of 70 eV, an ionisation current of 150 Μa and an ion source temperature of 200 °C [15]. In order to determine the Kovats index of the components, a mixture of alkenes (C_9_–C_24_) was added to the crude extract before injecting in the GC–MS equipment and analysed under the same conditions as above. The compounds were identified by computer searches in the commercial libraries of the National Institute of Standard and Technology (NIST) and by their Kovats retention indexes. 

## 4. Conclusions

Standardization of medicinal plants plays an important role in the development of pharmaceutical drugs from these plants because standardization establishes quality and identity profile of the plant for the purpose of overall quality assurance. The results obtained from this study are encouraging and will be used as a reference data for the standardization of *C. spectabilis* and formulations containing *C. spectabilis* leaf extract as a main ingredient. 

## Figures and Tables

**Table 1:** Minimum inhibitions concentration value of *Cassia spectabilis.*

Extraction time (Month)	MIC Value (mg/mL)
1 2 3	6.25 6.25 6.25
